# Hemodialysis Procedures for Stable Incident and Prevalent Patients Optimize Hemodynamic Stability, Dialysis Dose, Electrolytes, and Fluid Balance

**DOI:** 10.3390/jcm13113211

**Published:** 2024-05-30

**Authors:** Stefano Stuard, Christophe Ridel, Mario Cioffi, Alijana Trost-Rupnik, Konstantin Gurevich, Marija Bojic, Yerkebulan Karibayev, Nilufar Mohebbi, Wojciech Marcinkowski, Vlasta Kupres, Jelena Maslovaric, Alon Antebi, Pedro Ponce, Mamdouh Nada, Maria Eva Baro Salvador, Jaroslav Rosenberger, Tomas Jirka, Kira Enden, Volodymyr Novakivskyy, Daniela Voiculescu, Martin Pachmann, Otto Arkossy

**Affiliations:** 1 FME Global Medical Office, 61352 Bad Homburg, Germany; martin.pachmann@freseniusmedicalcare.com (M.P.); otto.arkossy@freseniusmedicalcare.com (O.A.); 2 FME Global Medical Office, 94260 Fresnes, France; christophe.ridel@freseniusmedicalcare.com; 3 FME Global Medical Office, 80133 Napoli, Italy; mario.cioffi@freseniusmedicalcare.com; 4 FME Global Medical Office, 1351 Brezovica pri Ljubljani, Slovenia; alijana.trost-rupnik@freseniusmedicalcare.com; 5 FME Global Medical Office, Saint Petersburg 194354, Russia; konstantin.gurevich@freseniusmedicalcare.com; 6 FME Global Medical Office, 75400 Zvornik, Bosnia and Herzegovina; marija.bojic@freseniusmedicalcare.com; 7 FME Global Medical Office, Almaty 050003, Kazakhstan; yerkebulan.karibayev@freniusmedicalcare.com; 8 FME Global Medical Office, 8002 Zurich, Switzerland; nilufar.mohebbi@freseniusmedicalcare.com; 9 FME Global Medical Office, 60118 Poznań, Poland; wojciech.marcinkowski@freseniusmedicalcare.com; 10 FME Global Medical Office, 49210 Zabok, Croatia; vlasta.kupres@freseniusmedicalcare.com; 11 FME Global Medical Office, 11000 Beograd, Serbia; jelena.maslovaric@freseniusmedicalcare.com; 12 FME Global Medical Office, Ra’anana 4366411, Israel; alon.antebi@freseniusmedicalcare.com; 13 FME Global Medical Office, 1750-233 Lisboa, Portugal; pedro.ponce@freseniusmedicalcare.com; 14 FME Global Medical Office, Riyadh 12472, Saudi Arabia; mamdouh.nada@freseniusmedicalcare.com; 15 FME Global Medical Office, 28760 Madrid, Spain; mariaeva.baro@freseniusmedicalcare.com; 16 FME Global Medical Office, 04011 Košice, Slovakia; jaroslav.rosenberger@freseniusmedicalcare.com; 17 FME Global Medical Office, 16000 Praha, Czech Republic; tomas.jirka@freseniusmedicalcare.com; 18 FME Global Medical Office, 00380 Helsinki, Finland; kira.enden@freseniusmedicalcare.com; 19 FME Global Medical Office, 02099 Kyiv, Ukraine; volodymyr.novakivskyy@freseniusmedicalcare.com; 20 FME Global Medical Office, 013682 Bucuresti, Romania; daniela.voiculescu@freseniusmedicalcare.com

**Keywords:** hemodialysis procedures, hemodynamic stability, dialysis dose, dialysis electrolyte balance, dialysis fluid balance

## Abstract

The demographic profile of patients transitioning from chronic kidney disease to kidney replacement therapy is changing, with a higher prevalence of aging patients with multiple comorbidities such as diabetes mellitus and heart failure. Cardiovascular disease remains the leading cause of mortality in this population, exacerbated by the cardiovascular stress imposed by the HD procedure. The first year after transitioning to hemodialysis is associated with increased risks of hospitalization and mortality, particularly within the first 90–120 days, with greater vulnerability observed among the elderly. Based on data from clinics in Fresenius Medical Care Europe, Middle East, and Africa NephroCare, this review aims to optimize hemodialysis procedures to reduce mortality risk in stable incident and prevalent patients. It addresses critical aspects such as treatment duration, frequency, choice of dialysis membrane, dialysate composition, blood and dialysate flow rates, electrolyte composition, temperature control, target weight management, dialysis adequacy, and additional protocols, with a focus on mitigating prevalent intradialytic complications, particularly intradialytic hypotension prevention.

## 1. Introduction

**Figure 1:**
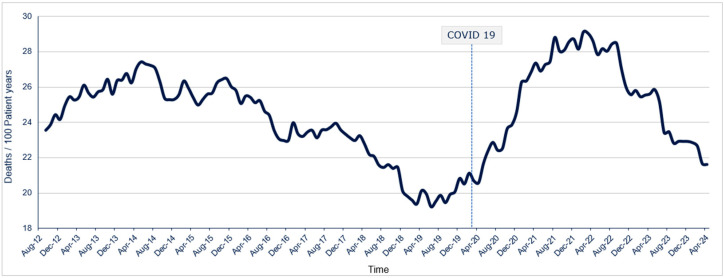
Incident patients’ mortality in Fresenius Medical Care EMEA NephroCare Clinics.

Worldwide, the number of patients with acute kidney injury, chronic kidney disease (CKD), and treated kidney failure has been steadily increasing for decades due to population aging and longer patient survival and is reported to exceed 850 million [1,2]. The demographic profile of patients with CKD transitioning to end-stage kidney disease (ESKD) and kidney replacement therapy (KRT) is evolving. The prevalence of aging patients with multiple comorbidities, such as diabetes mellitus and heart failure [3], undergoing hemodialysis (HD) is markedly higher than in the general population. It poses an increased challenge for healthcare providers [4,5]. Cardiovascular disease (CVD) stands as the foremost cause of mortality, significantly surpassing other causes [6,7,8]. This elevated mortality rate primarily stems from the substantial burden of underlying cardiovascular conditions [9]. Moreover, the cardiovascular stress imposed by the HD procedure further compounds this risk, exacerbating the already precarious health status of affected individuals [8,10]. The first year after transitioning from CKD to requiring HD has been associated with an increased risk of hospitalization and mortality, particularly during the first 90–120 days of HD initiation [11,12]. This vulnerability is particularly pronounced among the elderly population [13], even under usual circumstances. In addition, the early phase of HD is characterized by recurrent intradialytic complications. The COVID-19 pandemic created unprecedented challenges, particularly for susceptible populations such as patients with CKD. Dashtban et al. found that the mortality rate among CKD patients who initiated HD treatment during the COVID-19 pandemic was higher than the mortality rate among CKD patients who initiated HD treatment in the pre-pandemic period [14]. The lack of regular nephrology visits during the COVID-19 pandemic due to restrictions on in-person healthcare services or patients’ reluctance to seek medical care may result in inadequate management of comorbidities and complications associated with ESKD, thereby increasing mortality risk. In September 2014, Fresenius Medical Care (FME) Europe, Middle East, and Africa (EMEA) NephroCare implemented a new continuous quality improvement (CQI) policy called medical patient review CQI (MPR-CQI) in 20 countries [15]. MPR-CQI is based on medical key performance indicators (KPIs) related to patients’ clinical status and involves a process of evaluation, planning, and action aimed at improving patients’ clinical outcomes through monthly medical audits [15]. Supported by a digitalization process, the implementation of the MPR-CQI policy demonstrated a significant reduction in mortality [odds ratio 0.70 (95% confidence interval 0.65–0.76); *p* < 0.0001] by improving KPI target achievement in the post-implementation era [15]. However, a rise in mortality rates has been noted among CKD patients who initiated HD treatment (Figure 1). Considering the evolving demographics and clinical status of CKD patients transitioning to ESKD and HD and the reduction in patients starting HD treatment with arteriovenous fistula as the preferred vascular access, this review aims to improve HD procedures to mitigate intradialytic complications and mortality risk in stable incident (≤3 months in HD) and prevalent (>3 months in HD) patients by optimizing hemodynamic stability, dialysis dose, electrolytes, and fluid balance. The first section will delve into critical aspects such as intradialytic hypotension. The second will focus on treatment duration, frequency, choice of dialysis membrane, dialysate composition, and blood and dialysate flow rates. The subsequent segment will focus on dialysate electrolyte composition, temperature control, target weight management, dialysis adequacy, and additional protocols, primarily focusing on mitigating the prevalent intradialytic complications with specific attention to intradialytic hypotension prevention. The final segment focuses on intra- and extra-dialytic procedures, evaluating the impact of eating during dialysis, antihypertensive drugs, blood pressure monitoring, and the execution of blood tests.

## 2. Intradialytic Hypotension

The optimal medical definition for intradialytic hypotension (IDH) would identify a BP threshold below which individuals sustain end-organ pathologic insults with symptoms such as cramps, nausea, vomiting, and dizziness linked to adverse clinical outcomes [16]. However, despite its clinical significance, there is no consensus, evidence-based medical definition for the IDH condition [16]. Various definitions of IDH have been used over time: (1) K/DOQI Clinical Practice Guidelines, 2005: A decrease in systolic BP ≥ 20 mm Hg or a decrease in mean arterial pressure (MAP) ≥ 10 mm Hg associated with symptoms that include abdominal discomfort, yawning, sighing, nausea, vomiting, muscle cramps, restlessness, dizziness, or fainting; and anxiety [17]. (2) European Best Practice Guidelines (2007): A decrease in systolic BP ≥ 20 mm Hg or a decrease in MAP ≥ 10 mm Hg is associated with clinical events and the need for nursing interventions [18]. (3) UK Renal Association Guidelines (2009): An acute symptomatic fall in BP during dialysis requiring immediate intervention to prevent syncope [19]. (4) Japanese Society for Dialysis Therapy Guidelines (2012): A symptomatic sudden drop in systolic BP ≥ 30 mm Hg during dialysis or a decrease in the mean BP by ≥10 mm Hg [20]. In FME EMEA NephroCare, symptomatic IDH is defined as the fall of ≥20 mmHg in SBP from pre-dialysis to nadir intra-dialytic levels plus ≥2 responsive measures (stop treatment, move patient to the Trendelenburg position, IV infusion of liquids, reduction or stop of UF, reduction of the blood flow, etc.). Depending on the definitions, symptomatic IDH either during or immediately post-HD has been reported in 0.5% to 40% of all HD sessions [21,22,23], affecting 20–50% of HD patients [24]. More recent investigations indicate a prevalence of approximately 11% [3].

The multifaceted nature of IDH is underscored by the amplified incidence rates observed in a demographic characterized by advancing age and concomitant diseases, such as diabetes mellitus and heart failure [25]. While specific IDH episodes can be addressed and improved within clinical settings, others extend beyond the clinical environment and persist once the patient returns home.

Considering the potential risk low blood pressure imparts, IDH can no longer be treated as a benign condition. Chronic manifestations of IDH have been correlated with a spectrum of adverse outcomes, encompassing symptomatic discomfort, suboptimal dialysis, vascular access thrombosis, exacerbated renal function degradation, cardiovascular perturbations, and elevated mortality risks, predominantly attributed to recurrent organ compromise [6,26,27,28,29,30].

During HD, the fluid is removed from the intravascular compartment. The removal rate (ultrafiltration rate) may exceed that of refilling from the extracellular and intracellular compartments, reducing circulating blood volume. This can be magnified by the cardiopulmonary redistribution of blood flow that occurs when patients are dialyzed and dilatation of the capacitance vessels (dialysis thermal stress), leading to an increase in “unstressed blood volume” and a subsequent reduction in venous return [31]. In contrast to healthy persons, in whom a decline in plasma volume up to 15% (and in some cases up to 25%) is not associated with significant clinical features, IDH can occur at a much lower decline in blood volume [32]. This indicates that the normal compensatory response to hypovolemia can be disturbed in ESKD patients. IDH is exacerbated by a myriad of factors, including inter-dialytic fluid gains, CVD, antihypertensive medications, and the physiological demands placed on patients by HD. Hypotension can occur when one or more compensatory responses are incomplete: (A) reduced plasma refilling: rapid reduction of plasma osmolality in the first phase of dialysis treatment (e.g., consequent to urea removal) may result in movement of extracellular water into the cells [32]; (B) reduced sympathetic nervous system activation with failure of the acute hemodynamic compensatory response to hypovolemia; (C) reduced venoconstriction [33,34]; (D) reduced increase of peripheral arteriolar resistance [33,34]; and (E) reduced cardiac compensation due to reduced cardiac filling (preload), reduced myocardial contractility, reduced heart rate, and reduced cardiac output (stroke volume) [35].

## 3. Hemodialysis Procedures in Stable Incident and Prevalent Patients

FME EMEA NephroCare has created a set of HD strategies and procedures aimed at enhancing the management and seamless transition of stable incident patients with ESKD. This approach is designed to ensure meticulous equilibrium in fluid, electrolyte, and acid-base balance while concurrently maintaining hemodynamic stability during the HD procedure. Reducing HD-induced circulatory stress may necessitate combining various strategies customized to suit the unique characteristics of each patient’s inability to manage HD effectively [8]. Educating dialysis personnel to identify patients prone to IDH is relevant, facilitating the prompt recognition and prevention of such occurrences. Additionally, the recommendations advocate for an incremental approach to HD, underscoring the significant influence of residual renal function (RRF) on determining dosing schedules for intermittent HD. Starting HD treatment in stable incident patients may be challenging, as these patients are more likely to have multiple comorbidities and other complications that can make dialysis treatment difficult. The initial phase involves evaluating the patient’s overall health condition and recognizing possible risks or obstacles. This could entail conducting a comprehensive anamnesis review, performing a physical examination, and performing laboratory tests. It is crucial to closely observe the patient throughout and following dialysis sessions for any indications of complications. Physicians prescribing dialysis treatment to more complex and unstable incident patients, like those crashing in dialysis, should tailor the KRT according to the specific individual needs. This may involve choosing the type of dialysis (intermittent HD, continuous KRT, or peritoneal dialysis), the treatment frequency, and the dialysis machines’ settings. Below are suggested HD and dialysate prescriptions, ancillary laboratory tests, fluid status management, and intra- and extra-dialysis procedures that should be considered in stable incident and prevalent ESKD patients to improve their hard clinical outcomes.

### 3.1. Treatment Time and Frequency 

According to the European Best Practice Guideline (EBPG) on dialysis strategies [36] and the UK Renal Association Clinical Practice Guideline on Hemodialysis [37], prevalent patients should receive HD treatment at least three times per week, with a total duration of least at 12 h per week, unless significant RRF is present. Consideration should be given to increasing treatment time and/or frequency for patients experiencing frequent IDH, muscle cramps, headaches, and dizziness, as well as those who remain hypertensive despite maximum fluid removal and those with impaired phosphate control [36]. Increasing dialysis frequency has been shown to improve patient outcomes, with favorable effects on blood pressure control, nutritional status, hospitalization rates, and quality of life [36]. RRF should be periodically quantified in patients following an incremental dialysis schedule [37]. The National Kidney Foundation’s Kidney Disease Outcomes Quality Initiative (KDOQI) recommends that patients with low RRF (<2 mL/min) undergoing thrice weekly HD be prescribed a bare minimum of three hours per session [38]. Longer and/or additional HD treatments should be considered for patients with significant interdialytic weight gains, high UFR, who remain hypertensive, difficulty achieving dry weight, and with hyperphosphatemia, metabolic acidosis, and hyperkalemia [38]. A stepwise increase in treatment time and frequency should be considered according to RRF for stable incident patients not crashing on dialysis. These considerations are compactly presented in Table 1. 

### 3.2. Dialysis Membrane and Surface Area

The EBPG recommends using synthetic high-flux membranes to delay long-term complications of HD treatment like amyloidosis, to improve control of hyperphosphatemia, to reduce the increased cardiovascular risk, and to improve control of anemia [36]. The UK Renal Association Clinical Practice Guidelines on Hemodialysis recommend treating patients with minimal RRF with high-flux dialyzers [37]. KDOQI recommends using biocompatible, either high- or low-flux HD membranes for intermittent HD [38]. In FME EMEA, the dialyzer surface area standard is 1.6 ± 0.2 m^2^. Variations depend on individual patient needs and RRF. When initiating HD therapy in new patients, it is suggested to consider selecting high-flux, biocompatible dialyzers with smaller surface areas (Table 1). 

### 3.3. Blood Flow Rate

The blood flow rate (Qb) varies across regions and facilities. For instance, HD patients in the USA typically achieve a mean Qb greater than 400 mL/min, whereas in Japan, Qb is usually less than 200 mL/min [39,40]. Limited research exists on the correlation between Qb and hard clinical outcomes in HD patients. Consequently, the optimal Qb remains uncertain [40]. It is suggested to take into consideration a stepwise approach gradually from 150 mL/min in the first week of dialysis by a step of 50 mL/min over a week period to reach a Qb ≥ 340 mL/min in the 5th week of dialysis. Gradually increasing the Qb as patients become more accustomed to the HD process is reasonable (Table 1), ensuring individual tolerance depending on various factors, including the patient’s size, the type of vascular access, and individual metabolic patient needs. 

### 3.4. Dialysate Flow Rate

Like Qb, no single “best” dialysate flow rate (Qd) suits all HD patients. A Qd of 500 mL/min is commonly recommended for effectively conducting HD treatments. Increasing Qd from 500 to 800 mL/min has been suggested to enhance dialysis efficiency and permit shorter treatment durations. Leypoldt et al., through an in vitro study, showed that the improvement was attributed to better flow distribution within the dialysate compartment [41]. Modern dialyzers achieve improved dialysate fluid distribution through features such as hollow fiber undulations, spacer yarns, and changes in fiber packing density, which enhance flow distribution within the dialysate compartment. Albalate et al. investigated the effect of using Qd rates of 400, 500, and 700 mL/min with different modern dialyzers. They found that increasing Qd beyond 400 mL/min with these dialyzers offers limited benefits [42]. When comparing the authors’ differing recommendations regarding dialysate flow, a correlation emerges between the achieved simultaneous blood flow and assessing the impact of the dialysate flow [43]. If available, it is advisable to use autoflow methods that adjust the dialysate flow directly according to the blood flow. Compared to fixed dialysate flow, this approach can conserve resources while enhancing effectiveness. Increasing treatment duration represents a preferable alternative, providing demonstrated benefits to patients while reducing water consumption. It is suggested that Qd be gradually increased to 450–500 mL/min, maintaining a flow ratio (autoflow) of Qd/Qb = 1.2 for balanced dialysis performance. If the Kt/V falls below 1.4, consider raising Qd and autoflow beyond 500 and 1.2, respectively, if further increases in blood flow are not feasible and large dialyzer surface areas are utilized. Conversely, if HDF is used and the Kt/V exceeds 2.0, decrease the autoflow to ≤1.0 (Table 1).

## 4. Hemodialysis Procedures Aimed at Optimizing Hemodynamic Stability, Dialysis Dose, Electrolytes, and Fluid Balance

This is the third part of a review of HD procedures for stable incident and prevalent patients aimed at optimizing hemodynamic stability, dialysis dose, electrolytes, and fluid balance. Herein, we will focus on specific aspects of HD to minimize the risk of IDH in patients undergoing this treatment. We will discuss the importance of dialysate electrolytes, temperature regulation, target weight management, dialysis dose optimization, and other crucial procedures that play a key role in ensuring the safety and well-being of HD patients. By understanding and effectively implementing these strategies, healthcare providers can enhance the overall quality of care for individuals undergoing HD.

### 4.1. Dialysate Electrolytes 

Regular adjustment based on lab tests is critical, particularly for elements like potassium, bicarbonates, calcium, and sodium. Consistent monitoring can prevent dysregulation, which can cause complications like arrhythmias or bone disorders. In incident patients, a more frequent electrolyte evaluation is suggested in the first 30 days of HD treatment. A stepwise dialysate electrolyte prescription (Table 2) is suggested, and electrolytes in the dialysate should be regularly adjusted based on lab tests to prevent complications like arrhythmias or bone disorders.

#### 4.1.1. Dialysate Sodium 

Sodium balance is the cornerstone of intra-dialysis cardiovascular stability and reasonable inter-dialysis blood pressure control [44]. Acting on diffusive sodium (Na) mass transfer in terms of a reduced diffusive Na load may lead to a reduction of thirst [45], a reduction of interdialytic weight gain [46], and a modification of short-term outcomes thanks to reduced fluid overload and reduced blood pressure [47]. Sodium loading during HD results in greater thirst, increased volume expansion, increased cardiac workload, and subsequent hypertension [38]. While it is very reasonable to reduce the diffusive Na mass transfer in stable patients, particularly hypertensive patients with high interdialytic weight gain (IDWG), a cautious approach should be adopted in those with frequent IDH. In patients with minimal IDWG, the risk-benefit ratio may favor the use of higher dialysate Na (≥140 mEq/L) and regular reassessment to avoid prolonged Na loading and its consequences [48]. Low Na concentration in dialysate causes a decrease in plasma osmolarity that may lead to cellular overhydration, intradialytic cardiovascular instability with hypotension due to insufficient refilling of the intravascular compartment from the intracellular space, fatigue, muscle cramps, headache, and orthostatic hypotension [44,49]. A high dialysate Na concentration in the dialysate can prevent cardiovascular instability because of increased osmotic refilling of water from interstitial and intracellular to the intravascular compartments, which counteracts the effects of intravascular emptying secondary to ultrafiltration. The drawback is insufficient net Na removal, increased thirst, volume expansion, and hypertension [38,49]. Miskulin et al. demonstrated that using a dialysate Na 135 mEq/L, as opposed to 138 mEq/L, resulted in a slight reduction in IDWG without impacting IDH or pre-dialysis blood pressure, albeit with an increase in symptoms [50]. Conversely, raising the dialysate Na to 140 mEq/L decreased episodes of IDH despite a slight increase in IDWG and pre-dialysis blood pressure [50]. It is clear from the literature that there is no ideal dialysate Na. To minimize complications from both high and low dialysate Na, dialysis units typically choose a standard concentration between 138 and 140 mmol/L [51]. This range reflects likely optimization over time, as evidenced by 90% of DOPPS units adopting it for their standard D-Na [52]. The current practice of using a fixed dialysate Na concentration may not be ideal for personalized dialysis treatment. A more precise approach considers the dialysate-plasma Na gradient, which considers the patient’s pre-dialysis blood Na level [53]. This allows for a customized prescription based on individual needs [54]. Traditionally, this involved manually adjusting the dialysate Na to match the pre-dialysis plasma Na, requiring frequent monitoring [46,55]. However, new technology offers an automated Na balancing module that simplifies this process. With this tool, healthcare providers can easily tailor the dialysate Na concentration to both the patient’s Na level and the desired tonicity, improving treatment efficiency and, potentially, patient outcomes [56,57,58]. If the automated Na balance module is unavailable, a starting dialysate Na concentration of 140–143 mEq/L is suggested for patients initiating HD therapy. This selection prioritizes achieving initial cardiovascular stability during treatment by minimizing hemodynamic fluctuations caused by excessive Na gradients between the dialysate and plasma. The dialysate Na concentration may be progressively reduced to achieve a range of 138–140 mEq/L for prevalent patients (Table 2).

#### 4.1.2. Dialysate Bicarbonates 

The pre-dialysis serum bicarbonate should ideally fall within the range of 18.0–26.0 mmol/L [37]. Various factors can influence the measured bicarbonate concentration, including sample handling (e.g., blood exposure to air, which induces CO_2_ escape, resulting in low bicarbonate measurement) and analysis techniques. Bicarbonate concentration in dialysate in the range of 30–32 mEq/L may improve heart contractility by increasing ionized calcium [59]. Patients undergoing HD with elevated dialysate bicarbonate experience a rapid correction of metabolic acidosis, leading to an alkalotic state, particularly in the second half of treatment or afterward. This condition increases their risk of arrhythmias due to hypokalemia, hypocalcemia, hypomagnesemia, QT prolongation, vasodilation and hypotension, minute ventilation suppression, cerebral ischemia, and accelerated vascular calcification [60,61,62,63,64,65,66,67,68]. Tentori et al. demonstrated in a retrospective analysis of DOPPS data that clinics exposing patients to dialysate bicarbonate concentrations of 33–37 mEq/L experienced increased mortality, regardless of the blood bicarbonate concentration. Moreover, mortality rates were even higher when dialysate bicarbonate levels were 38 mEq/L or higher [69]. Another study found that reducing the dialysate bicarbonate concentration decreased IDH [70]. For patients starting HD therapy, a gentle correction of the uremic chronic acidosis status is suggested, beginning with a dialysate bicarbonate concentration ≤ 28 mEq/L. This approach prioritizes attaining initial cardiovascular stability during treatment while mitigating potential complications associated with rapid alkalization during HD and online HDF and subsequent alkalosis. In prevalent patients, the dialysate bicarbonate concentration may gradually increase to 30–32 mEq/L, adjusting it based on pre- and post-dialysis bicarbonate values and the patient’s metabolic status (Table 2). 

#### 4.1.3. Dialysate Calcium

The KDOQI goal range for corrected total serum calcium (Ca) should be between 8.4 and 10.2 mg/dL (2.1–2.54 mmol/L) [71]. Correction of acidosis and post-dialysis alkalosis may induce hypocalcemia, mainly if a low dialysate Ca concentration is used. Hypocalcemia can induce IDH through pro-arrhythmogenic effects [72,73,74]. In patients undergoing HD, prolongation of QTc is inversely correlated with intradialytic variations in plasma calcium, suggesting that patients with the most significant reduction in calcium had the greatest increases in QTc at the end of the HD session [69,70,71]. The KDIGO 2017 guidelines recommend a dialysate Ca between 1.25 and 1.5 mmol/L [75,76]. Dialysate Ca 1.25 mmol/L accelerates bone turnover and hemodynamic instability, resulting in a higher incidence of arrhythmia and sudden cardiac arrest [77,78], probably because of the increased QT interval [79]. Dialysate Ca 1.5 mmol/L improves cardiac muscle contractility and hemodynamic stability [10]. Dialysate Ca 1.75 mmol/L is associated with increased sympathetic activity, enhancing myocardial contractility, minimizing the decline in intradialytic blood pressure, and improving intradialytic hemodynamic stability [80,81]. Long-term effects contribute to accelerated vascular calcification and bone and mineral imbalance with adynamic bone [75,76,82,83,84]. In patients initiating HD therapy, the risk of IDH due to hypocalcemia’s pro-arrhythmogenic effects must be avoided. It is suggested that HD treatment be considered in incident patients with a dialysate Ca 1.5–1.75 mmol/L. This approach prioritizes achieving cardiovascular stability during treatment while mitigating potential hypocalcemia associated with rapid alkalization during HD and online HDF. For prevalent patients, the dialysate Ca may gradually decrease to a range of 1.25–1.5 mmol/L, adjusting it based on pre- and post-dialysis plasma calcium values. Maintaining an optimal calcium balance in ESKD patients is a complex process influenced by several factors, including plasma calcium levels, PTH, the use of phosphate binders and vitamin D analogs, and IDH risk and cardiac arrhythmias. These factors act in concert and require careful consideration for optimal outcomes. Therefore, dialysate Ca prescriptions should be carefully individualized [84].

#### 4.1.4. Dialysate Potassium

After a long interdialytic period, serum potassium (K) levels should be in the range of ≥4.0–≤6.0 mEq/L [37]. Potassium is important in maintaining the resting cell membrane potential, neuromuscular excitability, and cardiac pacemaker rhythmicity [85]. The HD treatment induces a sudden decrease in plasma K concentration during the first 60 min. In the last 60 min of HD, the plasma K concentration is stabilized, reaching a steady state during the last hour [86,87,88,89]. Lower dialysate K concentrations result in greater removal of K from the blood, leading to lower post-dialysis serum K levels [90]. The sudden decrease in plasma K concentration affects the intracellular and extracellular K concentration gradient, transmembrane potential, and repolarization of the cardiac cells. It can predispose to IDH, reducing cardiac output and consequently increasing the risk of arrhythmia, QT prolongation, and ectopic ventricular beats [91,92]. Hypokalemia during dialysis sessions has been correlated with brief paroxysmal atrial fibrillation episodes, most noted during the last two hours of dialysis [93,94]. Dialysate K < 2 mEq/L has been associated with cardiac events [95,96,97]. Strict control of K intake may decrease the need for low dialysate K, reducing the risk of intradialytic hypokalemia [98]. In patients initiating HD therapy, the risk of IDH due to hypokalemia’s pro-arrhythmogenic effects must be avoided. It is suggested to take into consideration starting the HD treatment in incident patients with a dialysate K = 3 mEq/L (Table 2). This approach prioritizes the achievement of cardiovascular stability during treatment while mitigating potential hypokalemia associated with rapid alkalization during HD and online HDF treatments. For prevalent patients, the dialysate K may be gradually decreased to a range of 2–3 mEq/L, adjusting it based on pre- and post-dialysis plasma potassium values (Table 2).

#### 4.1.5. Dialysate Magnesium

In ESKD patients, the ability to regulate magnesium (Mg) levels is impaired with increased accumulation risk, and studies have shown a link between high magnesium levels and IDH [99]. High Mg levels in ESKD patients can lead to excessive vasodilation and IDH. Imbalances in Mg levels may lead to changes in fluid compartments and exacerbate hypotension. Abnormal Mg levels can affect the heart’s electrical activity and potentially lead to irregular heart rhythms, possibly contributing to IDH. Kyriazis et al., investigating the effects of different dialysate Mg and Ca concentrations, reported that the combination with the fewest episodes of IDH was one of Mg 0.75 mmol/L and calcium 1.25 mmol/L, whereas dialysis solutions containing Mg 0.25 mmol/L and Ca 1.25 mmol/L triggered IDH due to an impairment of myocardial contractility [29,100]. In agreement with Floege, the commonly used dialysate magnesium concentration of 0.5 mmol/L (or rarely, 0.75 mmol/L) still appears acceptable pending further data [101,102] (Table 2). 

### 4.2. Dialysate Glucose

Both hypoglycemia and hyperglycemia should be avoided in ESKD patients to minimize the risk of IDH. Tight glycemic control and blood glucose monitoring are essential to maintaining hemodynamic stability during HD. Glucose-free dialysate could be utilized to avoid hypertriglyceridemia and the potential risk of increased bacterial growth in the dialysate [86]. However, this approach exposed patients to hypoglycemia in patients with diabetes treated with insulin [103,104]. A dialysate glucose concentration of 100 mg/dL is suggested [49].

### 4.3. Dialysate Temperature

Dialysate cooling has been demonstrated to increase hemodynamic stability by increasing systemic vascular resistance and enhancing cardiac contractility [105,106,107,108,109], whether through fixed reductions in dialysate temperature or isothermic dialysis. Using the fixed reduction method, the dialysate is cooled to 0.5–1.0 °C below the patient’s body temperature [110]. Using the isothermic method, the dialysate temperature is controlled via a biofeedback device that adjusts the dialysate temperature to prevent increased core body temperature, which generally occurs during HD [111]. Isothermic dialysis [112] is well tolerated and reduces the incidence of hypotension [113]. However, dialysate cooling can result in side effects such as chilling or cramps, particularly in patients prone to excessive vasoconstriction. Beneficial effects on multiple organ beds have been reported in short-term and longer-term observational studies and one randomized controlled study in incident dialysis patients [8,109,114]. Selby et al., in a randomized study of personalized cooling (MY TEMP), did not observe reductions in cardiovascular mortality or incidence of IDH [115]. Nevertheless, a recent large observational study suggests some patients may benefit from dialysate cooling [116]. Zoccali et al., by instrumental analyses, evaluated the correlation between dialysate temperature at the center level and the IDH incidence [116]. In this analysis conducted at the facility level with many adjustments for case-mix, with the same 0.5 °C lower dialysate temperature, the risk reduction for IDH occurrence was 33%, with a *p* < 0.001 significance; no association was found between mortality and temperature reduction [116]. In a recent review, Combe and Rubin [117] suggested that while dialysate cooling may not be a panacea solution to prevent IDH episodes [117], it may be efficient in certain patients [113,117,118] and, therefore, should be considered amongst various therapeutic measures for managing this condition [117]. In both incident and prevalent patients, dialysate cooling practices (fixed or isothermal) are suggested for individuals experiencing intradialytic hemodynamic instability. Lowering the dialysate temperature to 35.5 °C can reduce the risk of IDH [116] (Table 2). Ensure patients are comfortable and monitor for any symptoms of intolerance. 

### 4.4. Dry Body Weight 

The dry body weight (DBW) is the lowest tolerated post-dialysis weight achieved via a gradual change in post-dialysis weight at which there are minimal signs or symptoms of hypovolemia [119]. It refers to a patient’s weight after all excess fluid has been removed from the body through HD. DBW represents the patient’s most accurate weight without the extra fluid accumulating between dialysis sessions. Post-dialysis target weight should be regularly assessed because an inappropriately low target increases the risk of IDH [29,120,121]. DBW overestimation will increase the size of the interstitial space, minimizing the reduction of plasma volume during HD with elevated UF, but this may cause adverse effects related to an interstitial volume overload. Indeed, it is a significant risk factor in the development of hypertension, left ventricular hypertrophy, and CVD, therefore affecting the hard outcome risk of ESKD on HD treatment [122,123,124,125]. Underestimation of DBW can result in hypovolemia and can induce IDH, cramps, and dizziness [21]. Hypovolemia can lead to diminished blood perfusion to essential organs, resulting in frequent sub-ischemia and thereby potentially contributing to the decline of RRF [8]. There are several months between attaining DBW and adequate blood pressure control, termed the “lag phenomenon”, and this should be considered. The lag phenomenon reflects the slow stabilization of the extracellular fluid compartment while the chronic incident dialysis patient is converting from a catabolic to an anabolic state [126]. It is essential to establish an effective and accurate method for determining the DBW of ESKD patients in HD therapy. There are several methods to assess the DBW: Clinical Assessment (blood pressure weight trend, neck veins, peripheral edema, bioimpedance analysis, ultrasound lung comets (B-lines), ultrasound measurements of the inferior vena cava diameter, blood volume monitoring with or without biofeedback systems, laboratory parameters (B-type natriuretic peptides, low albumin level, high Ca125, soluble CD146) [29,127]. In FME EMEA NephroCare clinics, dry body weight, urea distribution volume, and fluid status are assessed using whole-body bioimpedance spectroscopy (BCM; FME) [125], as described by Moissl et al. [128] and Machek et al. [129]. BCM determines fluid overload (FO) in absolute liters independently of body composition by utilizing a physiological model based on normal tissue hydration [130]. Patients are overhydrated when their relative FO (calculated as FO divided by extracellular volume) is ≥15% in men and ≥13% in women, which corresponds to an absolute FO of about 2.5 L [124]. Tailoring DBW adjustments can improve cardiovascular stability during HD treatment. Slowly correcting the DBW and volume status for incident patients is suggested (Table 3). For prevalent patients, it is suggested that DBW and volume status be evaluated every 13 weeks based on clinical assessment and bioimpedance analysis, more frequently in cases of hospitalization or if requested (Table 3). 

### 4.5. Intradialytic Ultrafiltration

Intradialytic ultrafiltration, by removing excess fluid, enhances cardiac performance and venous oxygen saturation in ESKD patients. However, excessive ultrafiltration rates (UFR) pose a risk of myocardial and other organ ischemia. A lower UFR supports compensatory plasma refilling from the extracellular space. An increased treatment time slows the UFR and may help IDH-prone patients. The salt and water gains between dialysis treatments contribute to arrhythmic and hypotensive risks. Several studies have investigated the influence of UFR on patient outcomes. These studies suggest a potential link between higher UFR and the development of IDH, cardiovascular issues, and increased mortality rates [131,132,133,134,135,136]. The HEMO study demonstrated that a UF rate > 13 mL/kg/h was significantly associated with a greater hazard for the composite outcome of cardiovascular hospitalization and cardiovascular mortality. In contrast, UFR 10–13 mL/kg/h was not [132]. DOPPS found that a UFR > 10 mL/h/kg correlated with higher odds of IDH and a higher risk of all-cause mortality, while there was no association between UFR and cardiovascular mortality [133]. Flythe et al. observed a progressive increase in the risk of both overall mortality and cardiovascular mortality at UFRs exceeding 10 mL/h/kg [134]. The Kidney Care Quality Alliance established two quality measures focused on fluid management in HD. These measures prioritize avoiding UFR exceeding 13 mL/h/kg. This approach aims to reduce the incidence of IDH to minimize the risk of long-term damage to vital organs, including the heart, brain, and kidneys, ultimately improving patient survival rates [135]. Mermelstein et al. found a complex relationship between UFR, body weight, and mortality risk. Their study showed that the UFR linked to increased mortality risk varied depending on body weight and gender in high-body weight older patients and in high-vintage patients [136]. This highlights the importance of individualized treatment approaches in HD, considering factors beyond just body weight. For incident patients, a gradual adjustment of DBW is suggested based on clinical assessment and bioimpedance analysis. This approach allows for a more personalized and precise approach to fluid management for ESKD patients. It also helps to avoid UFR exceeding 10 mL/h/kg (Table 3). 

### 4.6. Dialysis Dose

For prevalent patients with minimal RRF undergoing HD three times per week, a single pool (sp) Kt/V ≥ 1.4 is recommended to ensure adequate dialysis adequacy [36,37] (Table 3). The urea distribution volume (V) should be measured using bioimpedance analysis. The dialysis dose may be reduced in patients with significant RRF, provided RRF is measured periodically to avoid inadequate dialysis [38]. For HD schedules other than thrice weekly, it is suggested to target a standard Kt/V of 2.3 volumes per week with a minimum delivered dose of 2.1 using a method of calculation that includes the contributions of ultrafiltration and RRF [38]. Convective Volume (Online HDF): Post-dilution online HDF has shown a direct effect in decreasing the incidence of IDH, improving hemodynamic stability—unrelated to improved Na+ balance [137,138,139], and positively impacting cardiac remodeling [140,141,142,143]. Five large randomized controlled trials (RCTs) have demonstrated the superiority of online HDF over high-flux dialysis in terms of hard clinical outcomes, particularly the survival rate of ESKD patients [144,145,146,147,148]. Studies suggest a convective volume above 23 L per session is most favorable for mortality rates [149,150,151,152,153]. Peters et al., in an individual patient data meta-analysis, combined four RCTs [144,145,146,147] and found a 14% reduction in all-cause mortality and a 23% reduction in cardiovascular mortality when treated with online HDF compared to high flux HD [154]. The most significant survival benefit was observed for patients receiving the highest delivered convection volume [154]. The CONVINCE study recently showed a reduction in the relative risk of all-cause mortality by 23%. This reduction was achieved through the prescription of post-dilution high dose (volume) HDF (HV-HDF), defined as convective volumes ≥ 23 L (range ± 1 L) per session [148].

For incident patients, it is recommended to follow a stepwise approach: progressively increase the substitution volume (QSub) from 5 L in the second week of dialysis, adding 5 L per week, aiming to reach a QSub of ≥21 L by the fifth week of dialysis (Table 3). QSub can be automatically prescribed to ensure optimal dialytic convective dose delivery based on changes in blood viscosity to prevent excessive hemoconcentration in the dialyzer, maximizing the filtration fraction based on transmembrane pressure. [155,156]. Concomitantly, the dialysate-bicarbonate concentration should also be progressively increased according to the metabolic characteristics of the patients. According to Canaud and Davenport, switching prevalent and stable dialysis patients to HV-HDF is more effortless than initiating a new patient in HDF [156]. They recommend starting with a post-dilution mode at 50 mL/min and gradually increasing by 25 mL/min per week to reach a convective volume of 125 mL/min. Consider switching to an automated convective-controlled mode once stable parameters are maintained [156].

## 5. Intra- and Extra-Dialytic Procedures 

### 5.1. Eating during HD

Food ingested before or during HD results in splanchnic blood pooling and the fall of systemic vascular resistance with the potential of IDH. Peripheral vascular resistance typically decreases 20 to 120 min following food intake, which may cause a decrease in blood pressure [157,158]. The risks associated with eating during HD include potential fluctuations in blood pressure, blood sugar levels, and electrolyte imbalances. These fluctuations can arise due to fluid and nutrient intake changes during the dialysis session, which may exacerbate existing health conditions or lead to adverse reactions. 

Therefore, incident patients who have started HD treatment should avoid eating for the next two weeks. Additionally, patients who experience frequent IDH should avoid eating during dialysis to maintain hemodynamic stability [159] (Table 4). On the other hand, this should be balanced against the risks of malnutrition and individualized according to the nutritional requirements and risk profile of patients for adverse intradialytic events [160,161]. 

### 5.2. Antihypertensive Drugs

Previous studies have demonstrated a U-shaped relationship between pre-dialysis systolic blood pressure (SBP) and clinical outcomes in dialysis patients [162,163,164]. Data from DOPPS suggests an optimal pre-dialysis SBP range of 130 to 160 mmHg [165]. Patients with very high pre-dialysis SBP (over 160 mmHg) experience a significantly increased risk of cardiovascular events and all-cause mortality [165]. Despite international guidelines recommending against using pre-dialysis blood pressure for diagnosing and managing hypertension in HD patients, many nephrologists continue to find it a simple and valuable tool in clinical practice [166]. Since 2014, FME EMEA NephroCare medical governance has implemented this approach, combining a pre-dialysis SBP target of 130–160 mmHg with regular fluid status evaluation and correction via bioimpedance monitoring every 13 weeks or more frequently in cases of clinical necessity or after hospitalization [125]. It’s crucial to be cautious with antihypertensive medications, as some might exacerbate the risk of IDH. As many as 50–90% of ESKD patients suffer from hypertension [167], and finding the balance between antihypertensive management and IDH risk can be a significant challenge due to fluid removal during dialysis [25]. Excessive fluid volume is a common cause of high blood pressure in dialysis patients, and the use of antihypertensive drugs needs careful management to avoid exacerbating IDH. If antihypertensive drugs are administered shortly before or during a dialysis session, they could potentially lower the patient’s blood pressure too much, thereby increasing the risk of IDH. This is particularly concerning when large volumes of fluid are removed during dialysis, as this can further decrease blood pressure. For this reason, clinical staff often need to adjust the timing and dosage of antihypertensive medications in dialysis patients. For instance, they might recommend taking these medications at night or after dialysis rather than before to help reduce the risk of IDH. By promoting urine production and fluid removal on non-dialysis days, diuretics can help to reduce overall fluid overload and the volume of fluid that needs to be removed during dialysis, which might reduce the risk of IDH. The 2006 NKF KDOQI Guidelines recommend using diuretics in patients with RRF [168]. Patients retain responsiveness to diuretics until their GFR falls below 5 mL/min/1.73 m^2^ [169]. It is suggested to consider a re-evaluation of antihypertensive medication or the administration of a dialysable drug before the HD session to attenuate its antihypertensive effect in hypotension-prone patients [170]. While decreasing the patients’ fluid volumes to reach the target weights, antihypertensive medication should be tapered or discontinued as their hypertension improves [171]. We suggest the omission of antihypertensive medication or the administration of a dialysable drug before the HD session in hypotension-prone patients (Table 4). It’s a good practice to use diuretics on non-dialysis days, especially in patients with RRF, to manage fluid status (Table 4). Cardioselective beta-blockers, angiotensin-converting enzyme inhibitors, and angiotensin receptor blockers have demonstrated significant cardioprotective effects among the various antihypertensive medications. They are often utilized to prevent heart disease, stroke, and other cardiovascular conditions. However, given the elevated prevalence of IDH in ESKD patients, it is crucial to exercise caution when prescribing these agents for their cardioprotective properties. Closely monitoring BP during and between HD sessions is necessary, especially following dose adjustments or changes in medication regimens and timing.

### 5.3. Blood Pressure Monitoring

Given the risk of IDH, frequent blood pressure monitoring during dialysis is essential. Monitoring blood pressure during dialysis is crucial to evaluating the efficacy of current antihypertensive medications and adjusting medication dosages if needed to ensure blood pressure stays within the target range for the duration of the HD procedure (Table 4).

### 5.4. Blood Parameters

Regular blood tests, especially during the initial weeks of HD, are crucial for adjusting treatment parameters and dialysate electrolyte prescriptions. A weekly review of the most critical blood parameters (hemoglobin, urea, sodium, potassium, calcium, and bicarbonate) is recommended in the first month. Monthly checks thereafter make sense, including all blood parameters typically evaluated in HD patients (Table 4).

## 6. Conclusions 

The first 90 days after starting HD treatment are generally considered critical because patients need to adapt physically, psychologically, and clinically to this new clinical status. Fresenius Medical Care EMEA NephroCare has developed a series of intra- and extra-HD procedures to improve the management and smooth transition of stable incident patients with ESKD and ensure the continuation of dialysis treatment in stable prevalent patients. This approach is designed to maintain meticulous fluid, electrolyte, and acid-base balance equilibrium while ensuring hemodynamic stability during the HD procedure. All presented intra- and extra-dialytic procedures are intended to minimize the risks of fluid overload, hypertension, and electrolyte disorders, as well as reduce the risk of intra- and extra-dialysis hypotension and cardiac arrhythmias.

We believe a more customized approach to HD, informed by risk stratification using artificial intelligence predictive and prescriptive models and incorporating genetic factors, holds immense potential to improve clinical outcomes for patients with ESKD. This approach will optimize treatment, improve patient care and quality of life, and reduce treatment-related side effects with prolonged life expectancy.

## Figures and Tables

**Table 1:** For stable incident patients not crashing on dialysis, a stepwise increase in treatment time and frequency based on residual renal function, dialyzer surface area, blood, and dialysate flows should be considered. Slowly increasing the blood flow in the first 10 min of dialysis is suggested.

	Hemodialysis Procedures in Stable Incident and Prevalent Patients					
Time	1st Week	2nd Week	3rd Week	4th Week	≥5th Week–3rd Months	≥3rd Month
Frequency (*n*)	≥2 *	≥3 *	≥3 *	≥3 *	≥3 *	≥3 *
Treatment time (min)	120–180	≤180	≤240	≤240	≥240 ^†^	≥240 ^†^
Dialyzer surface area (m^2^)	≤1.4	≤1.4	≤1.4	≤1.4	1.6 ± 0.2 *	1.6 ± 0.2 *
Qb (mL/min)	≤150	≤200	≤250	≤300	≥340 ^†^	≥340 ^†^
Qd (mL/min); Qd/Qb	300; 1.5	300; 1.5	400; 1.2	400; 1.2	≥500; ≥1.2 (if spKt/V < 1.4)	

^†^ Different targets according to patient clinical conditions and characteristics. * Increase the dialyzer surface area according to patient characteristics. Qb: blood flow. Qd: dialysate flow. sp: single pool.

**Table 2:** Stepwise dialysate electrolyte prescription for stable incident patients who are not crashing on dialysis and are prevalent patients.

	Dialysate Prescription in Stable Incident and Prevalent Patients					
Time	1st Week	2nd Week	3rd Week	4th Week	≥5th Week–3rd Months	≥3rd Month
Sodium (mEq/L)	140–143 *	140–142 *	140–141 *	139–140 *	138–140 *	138–140 *
Bicarbonate (mEq/L)	≤28 *	≤29 *	≤30 *	≤31 *	≤32 *	≤32 *
Calcium (mmol/L)	1.50–1.75 *				1.25–1.50 *	1.25–1.50 *
Potassium (mEq/L)	3 *	3 *	2–3 *	2–3 *	2–3 *	2–3 *
Magnesium (mmol/L)	0.5				0.5	0.5
Glucose (mg/dL)	100				100	100
Temperature (°C)	36.5 (≤36.0 in case of IDH)				36.0 (if IDH: ≤36.0 if tolerated)	

* Adjust the dialysate electrolyte prescription according to the patient’s blood test results. IDH: Intradialytic hypotension.

**Table 3:** Stepwise fluid status management and dialysis dose in stable incident patients not crashing on dialysis and prevalent patients.

	Fluid Status Management and Dialysis Dose in Stable Incident and Prevalent Patients					
Time	1st Week	2nd Week	3rd Week	4th Week	≥5th Week–3rd Months	≥3rd Month
DBW ^1^	Slowly correct				As week 1–4th	3 months *
Fluid status	Before 1st HD	Bi-weekly			Monthly	3 months *
UFR (mL/h/kg)	≤10 **	≤10 **	≤10 **	≤10 **	≤13 **	≤13 **
spKt/V	No targets				≥1.4	≥1.4
Post-dilution Qsub (L)	0	≤5	≤10	≤15	≥21 (Convective vol. ^2^ ≥23 L)	

^1^ DBW (dry body weight)/volume status should be evaluated by clinical assessment and instrumental methods. * Dry body weight/volume status evaluation should be repeated every 3 months or more frequently if requested, and mandatory after hospitalization. More frequently if requested and mandatory after hospitalization. ** Reduce in cases of frequent intradialytic hypotensive episodes. UFR: ultrafiltration rate. sp: single pool. Qsub: substitution volume. ^2^ Convective Vol.: Post-dilution convective volume is quantified as the sum of post-dilution Qsub (L) + ultrafiltration volume (L)/hemodiafiltration session.

**Table 4:** Stepwise approach to intra- and extra-dialytic procedures for incident and prevalent patients.

	Intra- and Extra Dialytic Procedures in Stable Incident and Prevalent Patients					
Time	1st Week	2nd Week	3rd Week	4th Week	≥5th Week–3rd Months	≥3rd Month
Eating during HD	Avoid	Avoid	Avoid in case of IDH		Avoid in case of IDH	
AHTs ^1^	Tapering while decreasing the body weight				Avoid non-dialyzable AHTs in case of IDH	
Diuretics	Only in interdialytic days if IDH				Only in interdialytic days if IDH	
RRF evaluation	Before 1st HD	No	No	No	Monthly *	Quarterly *
BP monitoring	Pre-Post HD and every 30 min **				Pre-Post HD and every 60 min **	
Blood tests ***	Before 1st HD	Weekly	Weekly	Weekly	Monthly	Monthly

^1^ AHTs: antihypertensive drugs. RRF: residual renal function. BP: blood pressure. * Applicable in cases of diuresis/die > 500 mL. ** More frequent in the case of intradialytic hypotension (IDH). ***: Hemoglobin, urea, calcium, bicarbonate, potassium, and sodium.
